# 1-Deoxynojirimycin Alleviates Liver Injury and Improves Hepatic Glucose Metabolism in *db*/*db* Mice

**DOI:** 10.3390/molecules21030279

**Published:** 2016-02-27

**Authors:** Qingpu Liu, Xuan Li, Cunyu Li, Yunfeng Zheng, Fang Wang, Hongyang Li, Guoping Peng

**Affiliations:** 1 College of Pharmacy, Nanjing University of Chinese Medicine, Nanjing 210023, China; lqpcy1224@163.com (Q.L.); xuanli@njutcm.edu.cn (X.L.); licunyuok@163.com (C.L.); zyunfeng88@126.com (Y.Z.), wangfa8875@163.com (F.W.); lihongyang@sina.com (H.L.); 2 Jiangsu Collaborative Innovation Center of Chinese Medicinal Resources Industrialization, Nanjing 210023, China

**Keywords:** 1-Deoxynojirimycin, *db*/*db* mice, liver, lipid metabolism, hepatic glucose metabolism, hepatic glycogen, glucose metabolic enzymes, hepatic insulin sensitivity, PKB/GSK-3β

## Abstract

The present study investigated the effect of 1-Deoxynojirimycin (DNJ) on liver injury and hepatic glucose metabolism in *db*/*db* mice. Mice were divided into five groups: normal control, *db*/*db* control, DNJ-20 (DNJ 20 mg·kg^−1^·day^−1^), DNJ-40 (DNJ 40 mg·kg^−1^·day^−1^) and DNJ-80 (DNJ 80 mg·kg^−1^·day^−1^). All doses were treated intravenously by tail vein for four weeks. DNJ was observed to significantly reduce the levels of serum triglyceride (TG), total cholesterol (TC), low density lipoprotein cholesterol (LDL-C) and liver TG, as well as activities of serum alanine aminotransferase (ALT), and aspartate transaminase (AST); DNJ also alleviated macrovesicular steatosis and decreased tumor necrosis factor α (TNF-α), interleukin-1 (IL-1), interleukin-6 (IL-6) levels in liver tissue. Furthermore, DNJ treatment significantly increased hepatic glycogen content, the activities of hexokinase (HK), pyruvate kinase (PK) in liver tissue, and decreased the activities of glucose-6-phosphatase (G6Pase), glycogen phosphorylase (GP), and phosphoenolpyruvate carboxykinase (PEPCK). Moreover, DNJ increased the phosphorylation of phosphatidylinositol 3 kinase (PI3K) on p85, protein kinase B (PKB) on Ser473, glycogen synthase kinase 3β (GSK-3β) on Ser9, and inhibited phosphorylation of glycogen synthase (GS) on Ser645 in liver tissue of *db*/*db* mice. These results demonstrate that DNJ can increase hepatic insulin sensitivity via strengthening of the insulin-stimulated PKB/GSK-3β signal pathway and by modulating glucose metabolic enzymes in *db*/*db* mice. Moreover, DNJ also can improve lipid homeostasis and attenuate hepatic steatosis in *db*/*db* mice.

## 1. Introduction

**Figure 1:**
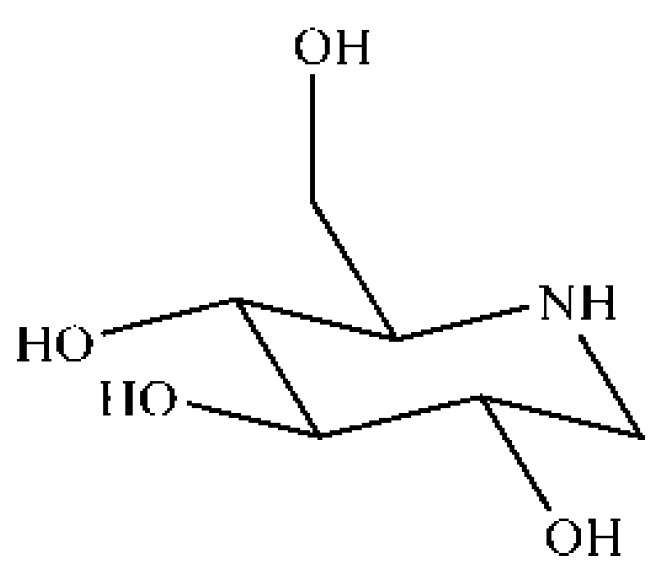
The chemical structure of 1-deoxynojirimycin.

Diabetes mellitus is a chronic disease and one of the world’s most serious health concerns. The incidence of type 2 diabetes is increasing around the globe [1]. Type 2 diabetes is mostly a defect that is characterized by high blood glucose due to insulin resistance in muscle, adipose, and liver [2,3]. However, pharmacological agents for type 2 diabetes exhibit a number of limitations, such as side effects and a high rate of secondary failure [4]. Thus, diabetic people and healthcare professionals are interested in natural products with a therapeutic potential on diabetes treatment, particularly those derived from plants because these sources are regarded to be less toxic with fewer side effects when compared to their synthetic counterparts. Mulberry leaves have been used in China and other Asian countries to treat diabetes based on reports of anti-diabetic effects in experimental animals [5,6,7]. The glucose analog 1-Deoxynojirimycin (DNJ), a potent α-Glucosidase inhibitor [8,9,10,11], is the most abundant iminosugar (Figure 1) in mulberry leaves, which was first isolated from the mulberry leaves (*Morus alba* L.) by Yagi and coworkers [12].

In our previous study, we have demonstrated that DNJ could alleviate hyperglycemia by improving skeletal muscle insulin sensitivity in *db*/*db* mice [13]. Type 2 diabetes is a multifactorial disease, characterized by hyperglycemia, insulin deficiency, and insulin resistance [14]. The liver is an insulin-sensitive organ that regulates energy homeostasis and plays a major role in whole body glucose metabolism by maintaining a balance between glucose production and glucose storage in the form of glycogen [15,16]. In a diabetic state, the liver becomes resistant to the actions of insulin and there is an increase in hepatic glucose production, which is a major contributor to hyperglycemia [15]. We have showed that DNJ could improve skeletal muscle insulin sensitivity [13], however, it is not clear whether DNJ can improve hepatic insulin sensitivity in *db*/*db* mice. Additionally, *db*/*db* mice are an insulin resistant diabetes animal model accompanied by obesity. Obesity always impairs hepatic glucose and lipid homeostasis, and this metabolic dysregulation can ultimately cause hepatic steatosis [17,18,19]. Notably, hepatic steatosis, insulin resistance, and obesity occur frequently and simultaneously, and the presence of these conditions synergistically increases the risk of type 2 diabetes [20].

Therefore, the aim of the present study was to investigate the effects of DNJ on the liver of *db*/*db* mice. Additionally, “could DNJ improve hepatic glucose metabolism and hepatic insulin sensitivity in *db*/*db* mice or not?”, and “could DNJ attenuate hepatic steatosis and improve lipid homeostasis in *db*/*db* mice”, were questions that were posed.

## 2. Results

### 2.1. Serum Levels of Triglyceride (TG), Total Cholesterol (TC), High Density Lipoprotein Cholesterol (HDL-C) and Low-Density Lipoprotein Cholesterol (LDL-C)

**Figure 2:**
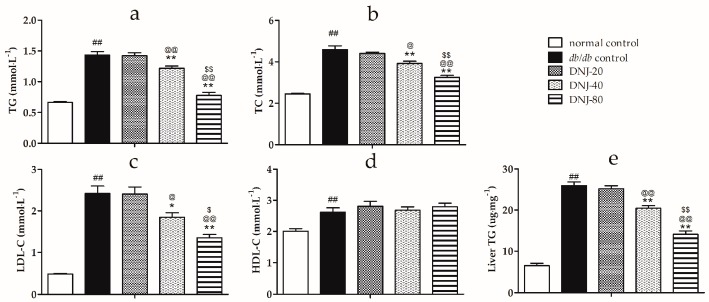
Effect of DNJ on the levels of serum triglyceride (TG) (**a**); total cholesterol (TC) (**b**); low density lipoprotein cholesterol (LDL-C) (**c**); high density lipoprotein cholesterol (HDL-C) (**d**) and liver TG content (**e**) in *db/db* mice. ^##^ *p* < 0.01 *vs.* normal control. * *p* < 0.05, ** *p* < 0.01 *vs.* *db/db* control, ^@^ *p* < 0.05, ^@@^ *p* < 0.01 *vs.* DNJ-20, ^$^ *p* < 0.05, ^$$^ *p* < 0.01 *vs.* DNJ-40 (*n* = 6).

As shown in Figure 2, the serum levels of triglyceride (TG), total cholesterol (TC) and low-density lipoprotein cholesterol (LDL-C) were significantly higher in *db*/*db* mice than in the normal control group (*p* < 0.01). DNJ treatment remarkably reduced serum levels of TG, TC and LDL-C in a dose-dependent manner. The high density lipoprotein cholesterol (HDL-C) level in *db*/*db* mice was also elevated, and there was no change after treatment with DNJ. Moreover, DNJ treatment tended to lower the liver TG content in a dose-dependent manner in *db*/*db* mice (Figure 2e).

### 2.2. Effect of CGB on Activities of Alanine Aminotransferase (ALT) and Aspartate Transaminase (AST)

**Figure 3:**
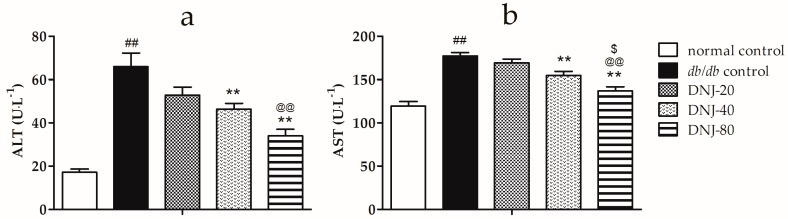
Effects of DNJ on activities of serum alanine aminotransferase (ALT) (**a**) and aspartate transaminase (AST) (**b**) in *db*/*db* mice. ^##^ *p* < 0.01 *vs.* normal control. ** *p* < 0.01 *vs.* *db/db* control, ^@@^ *p* < 0.01 *vs.* DNJ-20, ^$^ *p* < 0.05 *vs.* DNJ-40 (*n* = 6).

To investigate the effect of DNJ on hepatic function, serum alanine aminotransferase (ALT) and aspartate transaminase (AST) activities were determined. As shown in Figure 3, the activities of ALT and AST were significantly higher in *db*/*db* groups than in normal control group (*p* < 0.01). However, the activities of ALT and AST were notably decreased in DNJ-treated groups compared to the *db*/*db* control group.

### 2.3. Effects of DNJ on Hepatic Pathological Changes

**Figure 4:**
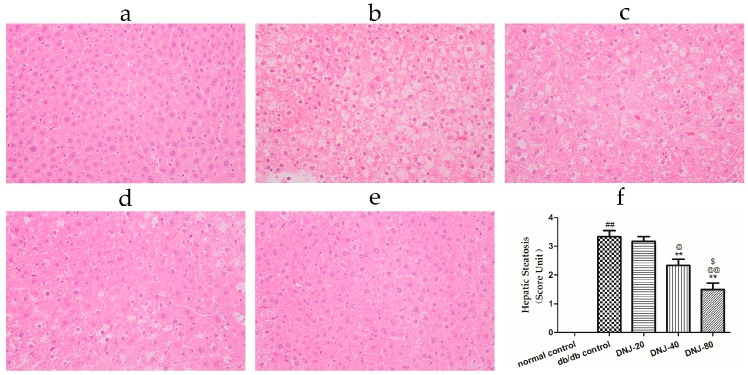
Effect of DNJ on hepatic pathological changes. Histological observation of the hematoxylin-eosin (H & E) sections (original magnification ×200). Macrovesicular steatosis was observed in the livers of *db*/*db* mice. (**a**) normal control; (**b**) *db*/*db* control; (**c**) DNJ-20; (**d**) DNJ-40; and (**e**) DNJ-80; additionally, quantification of hepatic steatosis (**f**) were carried out. ^##^ *p* < 0.01 *vs.* normal control. ** *p* < 0.01 *vs.* *db/db* control, ^@^ *p* < 0.05, ^@@^ *p* < 0.01 *vs.* DNJ-20, ^$^ *p* < 0.05 *vs.* DNJ-40 (*n* = 6).

Morphological changes of the liver are shown in Figure 4; lipid accumulation in the liver resulted in a pale discoloration (Figure 4b), lipid vacuoles occupied much of the hepatocyte cytoplasm, the nucleus and other organelles were pushed to the periphery of the cell, and some hepatocytes appeared bloated. Liver lipid content and lipid droplet size were significantly attenuated by DNJ treatment in a dose-dependent manner.

### 2.4. Effects of DNJ on Levels of Tumor Necrosis Factor α (TNF-α), Interleukin-1 (IL-1) and Interleukin-6 (IL-6) in Liver Tissue

**Figure 5:**
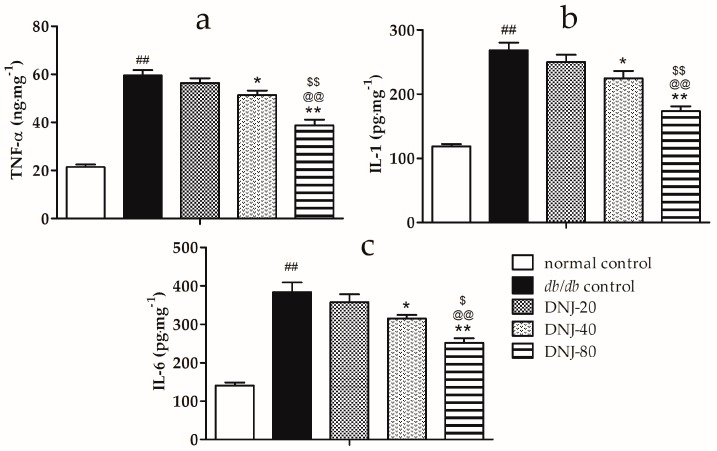
Effects of DNJ on the expression of tumor necrosis factor α (TNF-α) (**a**); interleukin-1 (IL-1) (**b**); and interleukin-6 (IL-6) (**c**) in liver tissue of *db*/*db* mice. ^##^ *p* < 0.01 *vs.* normal control. * *p* < 0.05, ** *p* < 0.01 *vs.* *db/db* control, ^@@^ *p* < 0.01 *vs.* DNJ-20, ^$^ *p* < 0.05, ^$$^ *p* < 0.01 *vs.* DNJ-40 (*n* = 6).

Hepatic steatosis is always associated with inflammation. To explore the effects of DNJ on inflammation, we conducted ELISA experiments to analyze hepatic tumor necrosis factor α (TNF-α), interleukin-1 (IL-1), and interleukin-6 (IL-6) levels in the liver tissue of *db*/*db* mice. As shown in Figure 5, the hepatic TNF-α, IL-1, and IL-6 content of *db*/*db* groups were significantly higher than those of the normal control group (*p* < 0.01). However, DNJ treatment notably decreased the levels of TNF-α, IL-1, and IL-6 in liver tissue in a dose-dependent manner when compared with the *db*/*db* control group.

### 2.5. Effects of DNJ on Hepatic Glycogen Content, Activity Level of Hexokinase (HK), Pyruvate Kinase (PK), Glycogen Phosphorylase (GP) and Glucose-6-Phosphatase (G6Pase)

In our previous study, we have showed that DNJ could decrease the levels of glucose and insulin in a dose dependent manner, in the range of 20–80 mg·kg^−1^·day^−1^ [13]. In the present study, we mainly focused on hepatic glucose metabolism, and the livers used in this study were from the same cohorts of mice used in the previous work.

**Figure 6:**
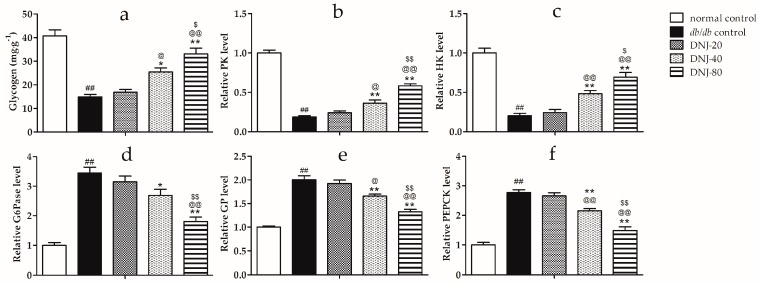
Effect of DNJ on hepatic glycogen content in *db/db* mice (**a**) and levels of pyruvate kinase (PK) (**b**); hexokinase (HK) (**c**); glucose-6-phosphatase (G6Pase) (**d**); phosphorylase (GP) (**e**); and phosphoenolpyruvate carboxykinase (PEPCK) (**f**) in the liver tissue of *db/db* mice. ^##^ *p* < 0.01 *vs.* normal control. * *p* < 0.05, ** *p* < 0.01 *vs.* *db/db* control, ^@^ *p* < 0.05, ^@@^ *p* < 0.01 *vs.* DNJ-20, ^$^ *p* < 0.05, ^$$^ *p* < 0.01 *vs.* DNJ-40 (*n* = 6).

As shown in Figure 6a, hepatic glycogen content in the *db*/*db* groups was significantly lower than that in the normal control group (Figure 6a). After being treated by DNJ, hepatic glycogen content in *db*/*db* mice notably increased in a dose-dependent manner.

Furthermore, hexokinase (HK) and pyruvate kinase (PK) activities were significantly lower in *db*/*db* mice compared with the normal control mice (Figure 6b,c); however, in the DNJ treated *db/db* mice, the activities of these enzymes were markedly increased in a dose-dependent manner when compared with the *db*/*db* control. Meanwhile, glucose-6-phosphatase (G6Pase), glycogen phosphorylase (GP), and phosphoenolpyruvate carboxykinase (PEPCK) activities were notably higher in *db*/*db* mice compared to the normal control mice (Figure 6d–f), and these enzymes’ activities were remarkably decreased in a dose-dependent manner after being treated using DNJ. These results suggest that DNJ can normalize the activities of these five pivotal enzymes for glucose metabolism to maintain glucose homeostasis in the liver tissue of *db*/*db* mice.

### 2.6. Effect of DNJ on Insulin PKB/GSK-3β Signal Pathway

**Figure 7:**
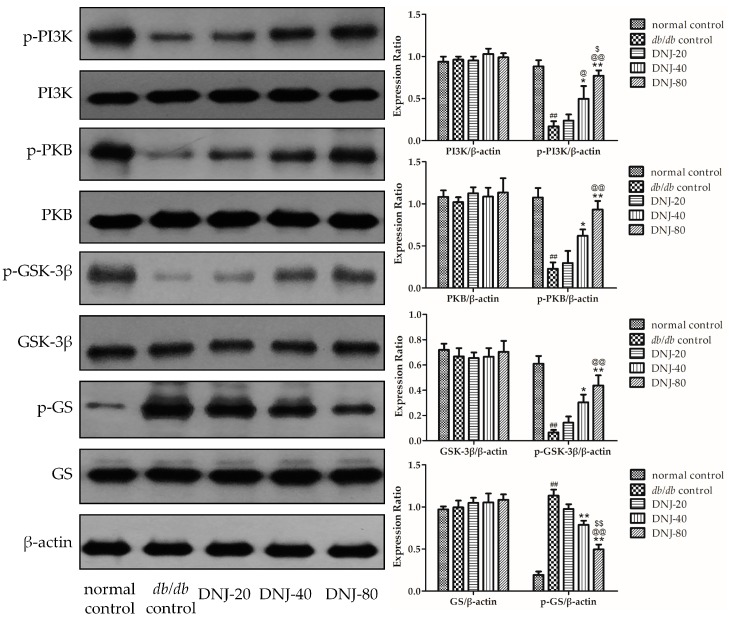
Effect of DNJ on PKB/GSK-3β signal pathway in the liver of *db/db* mice. Expressions of phosphatidylinositol 3 kinase (PI3K), protein kinase B (PKB), glycogen synthase kinase 3β (GSK-3β), glycogen synthase (GS) and their phosphorylation were determined. ^##^ *p* < 0.01 *vs.* normal control. * *p* < 0.05, ** *p* < 0.01 *vs.* *db/db* control, ^@^ *p* < 0.05, ^@@^ *p* < 0.01 *vs.* DNJ-20, ^$^ *p* < 0.05, ^$$^ *p* < 0.01 *vs.* DNJ-40 (*n* = 3).

To investigate whether insulin-stimulated PKB/GSK-3β signal pathway might be responsible for the effects of DNJ on liver insulin sensitivity in *db*/*db* mice, we conducted Western blot experiments to analyze the expression levels of phosphatidylinositol 3 kinase (PI3K), protein kinase B (PKB), glycogen synthase kinase 3β (GSK-3β), glycogen synthase (GS) and their phosphorylation. As shown in Figure 7, there were no differences in the total PI3K, PKB, GSK-3β, and GS protein expressions, but the phosphorylation of p85-PI3K, Ser473-PKB, and Ser9-GSK3β in liver tissue were notably lower, and the phosphorylation of Ser645-GS was significantly higher in the *db*/*db* control group than in the normal control group (*p* < 0.01). After being treated with DNJ for four weeks, despite decreased fasting insulin levels [13], the phosphorylation of p85-PI3K, Ser473-PKB, and Ser9-GSK3β were obviously increased and the phosphorylation of Ser645-GS was notably decreased when compared with *db*/*db* control mice.

## 3. Discussion

In our previous study, we have showed that DNJ could decrease the levels of glucose and insulin via increasing skeletal muscle insulin sensitivity [13]. In this study, we mainly focused on the effects of DNJ on the liver of *db*/*db* mice. We found that DNJ could also improve hepatic glucose metabolism and increase hepatic insulin sensitivity. Moreover, the present study showed that DNJ could improve lipid metabolism and alleviate hepatic steatosis in *db*/*db* mice.

Compared with wild-type C57BLKS mice, *db*/*db* mice developed a stably higher serum TC, TG, and LDL-C, and DNJ treatment effectively decreased these biochemical indexes. Furthermore, DNJ treatment also significantly lowered the liver TG content when compared with the *db*/*db* control. However, the HDL-C levels in *db*/*db* mice were also elevated, which is in line with the study by Zheng and coworkers [21]; this is different from humans who are obese or have diabetes. Several studies have shown that DNJ-rich mulberry leaf extract has been used to potentially improve lipid profiles in a human study [22] and to suppress lipid accumulation *in vivo* [23]. These results indicate that DNJ could improve lipid metabolism. Moreover, it was reported that mulberry leaf extract protects damaged liver against HFD-induced non-alcoholic fatty liver disease (NAFLD), demonstrated by a hepatic function test (AST and ALT levels) [24]. AST and ALT are the key indicators of liver function and our study showed that DNJ decreased the activities of serum ALT and AST, combined with an attenuating degree of liver steatosis. These results implied that DNJ has a protective effect for liver in *db*/*db* mice.

Furthermore, DNJ treatment significantly decreased hepatic TNF-α, IL-1, and IL-6 levels in *db*/*db* mice. TNF-a, IL-1, and IL-6 play critical roles in the progression of liver injury, which provoke hepatocellular injury and death [25,26,27]. TNF-α and IL-1 are sensitizing factors, acting on leukocyte infiltration of the liver and the effect of these cytokines and chemokines amplified hepatocyte damage [28,29]. IL-6 is a pro-inflammatory cytokine that has been proposed to have a direct and indirect deleterious role in induction inflammation [30]. Wieckowska and coworkers demonstrated a markedly increased hepatic IL-6 expression assayed with immunohistochemistry in patients with non-alcoholic steatohepatitis, as compared to normal liver, and found hepatic IL-6 expression to be correlated with the severity of inflammation and fibrosis [31]. It is reported that mulberry water extract treatment down-regulated the expression of TNF-α and IL-6 to inhibit pro-inflammatory mediators in alcohol-induced liver injury [32]. In the present study, our results demonstrated that the anti-inflammatory effect may partly contribute to the liver-protective effect of DNJ in *db*/*db* mice.

Glycogen is an important glycometabolism index and glycogen synthesis plays an important role in glucose homeostasis and glycometabolism [33,34]. Glycogen synthesis disorder is a major symptom of type 2 diabetes [35,36,37,38]. Some studies have confirmed that hepatic glycogen level was decreased in insulin resistant animal models [21,39]. In this study, DNJ treatment markedly increased the low hepatic glycogen content in *db*/*db* mice. The results demonstrated that DNJ can increase hepatic glycogen storage. Furthermore, we investigated the key enzymes of glycometabolism, including HK, PK, GP, G6Pase, and PEPCK in liver tissue. HK and PK play important roles in the utilization of blood glucose in glycolysis for energy production or in glycogen reserves in the liver [40,41,42,43,44]. GP, G6Pase, and PEPCK play critical roles in gluconeogenesis reactions, and confer on the liver the capacity to release glucose into the blood [34,45]. Decreased HK and PK, and increased GP, G6Pase, and PEPCK activities have been confirmed in several animal models of diabetes [46,47,48,49]. It was reported that DNJ increased hepatic PK activities and decreased hepatic PEPCK activities in streptozotocin (STZ) Induced Diabetic Mice [50]. Liu and coworkers reported that branch bark extract of mulberry treatment decreased G6Pase mRNA expression in the liver of STZ-Induced Diabetic Mice [51]. Bollen and coworkers reported that DNJ decreased the rate of hepatic glycogenolysis induced by glucagon in normal Wistar rats [52]. In the present study, DNJ treatment increased PK and HK activities in *db*/*db* mice, thereby increasing glycolysis in liver tissue, and reducing GP, G6Pase, and PEPCK activities in *db*/*db* mice, thereby decreasing gluconeogenesis. Consequently, DNJ-increased hepatic glycogen storage may be partly controlled through enhanced PK and HK activities and inhibited GP, G6Pase, and PEPCK activities.

Moreover, in order to gain insight into the related molecular mechanisms of DNJ increases in hepatic glycogen synthesis in *db*/*db* mice, we investigated insulin-stimulated PKB/GSK-3β pathway in the liver tissue of *db*/*db* mice. Glycogen synthesis in liver is regulated by GSK-3β, which regulates the enzyme GS, a key regulatory step in glycogen synthesis for glucose storage [53,54,55,56], while phosphorylation of GSK-3β on Ser9 inhibits GSK-3β activity, which leads to reduced phosphorylation and subsequent activation of glycogen synthase, thus increases glycogen synthesis [57,58]. In addition, GSK-3β is regulated upstream by PKB and PI3K [59,60]. Therefore, we investigated the expression of PKB, PI3K, GSK-3β, GS, and their phosphorylation. Bollen and coworkers reported that DNJ could inhibit the rate of hepatic glycogenolysis in the liver induced by glucagon, but this effect was not via affecting the concentration of phosphorylase α in normal Wistar rats [52]. In the present study, although there were no differences in the total PKB, PI3K, GSK-3β, and GS expressions among all groups, DNJ treatment increased phosphorylation of p85-PI3K and Ser473-PKB, and subsequently promoted phosphorylation of Ser9-GSK3β, which correlated with decreased phosphorylation of Ser645-GS and increased GS activity in *db*/*db* mice. Combined with the decreased fasting insulin levels [13], these results demonstrate that DNJ improves hepatic glucose metabolism in *db*/*db* mice by increasing hepatic insulin sensitivity via strengthening the insulin-stimulated PKB/GSK-3β signaling pathway.

## 4. Materials and Methods

### 4.1. Materials

TG and TC commercial kits were purchased from Zhejiang Dongou Diagnostics Co., Ltd., (Wenzhou, China). HDL-C, LDL-C, AST, and ALT commercial kits were purchased from Nanjing Jiancheng Bioengineering Institute (Nanjing, China). TNF-α, IL-1, and IL-6 commercial ELISA kits were provided by Nanjing Jiancheng Bioengineering Institute (Nanjing, China). Glycogen content, HK, PK, and PEPCK commercial kits were purchased from Nanjing Jiancheng Bioengineering Institute (Nanjing, China). GP and G6Pase commercial ELISA kits were provided by Jiangsu Yutong Biological Technology CO., Ltd. (Yancheng, China). Bicinchonininc acid protein assay was purchased from Beyotime Institute of Biotechnology (Nanjing, China). PI3K, PKB, p-Ser473-PKB, and β-actin antibodies were provided by Cell Signaling Technology (Danvers, MA, USA). p-p85-PI3K antibody was a product of ABCAM (Cambridge, UK). GSK-3β, p-Ser9-GSK-3β, GS, and p-Ser645-GS were purchased from EnoGene (New York, NY, USA). HRP conjugated antibody IgG was provided by ABCAM. An Electro-Chemi-Luminescence (ECL) detection kit was purchased from Thermo Electron Corp., (Rockford, IL, USA).

### 4.2. Preparation of DNJ

Preparation of DNJ was performed as previously described [13]. Briefly, the dried mulberry leaves were extracted with boiling water (two times) for 1 h, the extract water was adjusted to pH 3–4 by using 37% HCl (*v*/*v*), and passed over a cation exchange resin (001 × 7) column. The cation exchange resin was eluted with ethanol–water (70:30, *v*/*v*), then eluted with ammonia (4%, *v*/*v*). Next, the ammonia effluent was gathered and subjected to an anion exchange resin (201 × 4) column, eluted by water. Then, the product was subjected to silica gel H column and eluted with 95% ethanol, and the eluent and concentrate were stored under vacuum. Finally, DNJ was prepared after being crystallized in 95% ethanol.

### 4.3. Animals

Male wild-type C57BLKS mice and C57BLKS/*Lepr*^db^ (*db/db*) mice were purchased from Model Animal Research Center of Nanjing University (MARC; Nanjing, China) at the age of 9th week. All the animals were maintained under standard light (12 h light/12 h dark) and temperature conditions (22 ± 2 °C). The mice were fed a standard chow diet and mice had free access to food and water *ad libitum*. All experimental procedures were performed in accordance with International Guidelines for Care and Use of Laboratory Animals, and were approved by the Animal Ethics Committee of Nanjing University of Chinese Medicine.

### 4.4. Experimental Design

At the end of 10th week age, all the mice were divided into five groups (*n* = 6):

Group 1 (normal control): wild-type C57BLKS mice were treated intravenously with normal saline through the tail vein.

Group 2 (*db/db* control): *db/db* mice were treated intravenously with normal saline by tail vein.

Group 3 (DNJ-20): *db/db* mice were treated intravenously with a DNJ dose of 20 mg·kg^−1^·day^−1^ through the tail vein.

Group 4 (DNJ-40): *db/db* mice were treated intravenously with a DNJ dose of 40 mg·kg^−1^·day^−1^ through the tail vein.

Group 5 (DNJ-80): *db/db* mice were treated intravenously with a DNJ dose of 80 mg·kg^−1^·day^−1^ through the tail vein.

Generally, the solution injected was warm and sterile. All the mice climbed into the mice holder themselves, and the tails of the mice were put into warm water, then the mice received tail vein intravenous injections around the proximal 1/3 of tail. The injection needles were changed in order to keep the injection needle from becoming dulled after several uses. All of these animal handling procedures were very gentle and soft. All experimental procedures were performed in accordance with International Guidelines for Care and Use of Laboratory Animals, and were approved by the Animal Ethics Committee of Nanjing University of Chinese Medicine. All doses were given for four weeks. At the end of the 14th week age, the mice were anesthetized with chloral hydrate after withholding food for 12 h, and blood samples were taken for biochemical estimations. Additionally, liver tissues were removed (after the blood was collected), then rinsed with a physiological saline solution, and immediately stored at −80 °C for Western blot analysis.

### 4.5. Biochemical Estimations

Serum levels of triglyceride (TG), total cholesterol (TC), high-density lipoprotein cholesterol (HDL-C), low-density lipoprotein cholesterol (LDL-C), aspartate transaminase (AST), alanine transaminase (ALT), and liver TG contents were determined using commercial kits. All commercial kits were used following manufacturer’s instructions.

### 4.6. Histological Examination

Liver tissues were formalin-fixed and embedded in paraffin. Sections (4-μm thick) were stained with hematoxylin-eosin (H & E) (Nanjing Jiancheng Bioengineering Institute, Nanjing, China) and examined using a light microscope (Olympus Medical Systems Corp., Tokyo, Japan). The degree of lipid infiltration on H & E staining was scored on a scale of 0–4, with 0 being normal healthy tissue and 4 being the worst, as described previously [61].

### 4.7. Determination of TNF-α, IL-1, and IL-6 in Liver Tissues

Inflammatory cytokines, including TNF-α, IL-1, and IL-6 levels in the liver tissues, were determined using commercial ELISA kits (Nanjing Jiancheng Bioengineering Institute, Nanjing, China). All commercial kits were used according to the manufacturer’s instructions.

### 4.8. Determination of Hepatic Glycogen and Enzymes of Glucose Metabolism in Liver Tissue

Glycogen content and the activities of HK, PK, and PEPCK in the liver tissue were measured using commercial kits (Nanjing Jiancheng Bioengineering Institute, Nanjing, China). The activities of GP and G6Pase in the liver tissue were measured using commercial ELISA kits (Jiangsu Yutong Biological Technology CO., Ltd., Yancheng, China). All commercial kits were used following manufacturer’s instructions.

### 4.9. Western Blot

In order to investigate the effects of DNJ on insulin-stimulated PKB/GSK-3β signaling pathways, Western blot analyses were performed as previously described [62]. Total protein was extracted using protein lysis buffer and protein concentration was quantified using bicinchonininc acid protein assay. Briefly, liver tissues (0.1 g) were lysed in lysis buffer (50 mM Tris (pH 7.4), 0.1% SDS, 150 mM NaCl, 1% NP40, 0.5% sodium deoxycholate, 1 μL protease inhibitor, 10 μL phosphatase inhibitors and 5 μL 100 mM PMSF), and centrifuged at 16,000× *g* at 4 °C for 15 min. Equal amounts of protein (70 μg) were loaded on 10% SDS-PAGE and transferred onto polyvinylidene fluoride (PVDF) membranes. After the membranes were blocked, they were incubated with primary antibodies against PI3K, *p*-p85-PI3K, PKB, *p*-Ser473-PKB, GSK-3β, *p*-Ser9-GSK-3β, GS, *p*-Ser645-GS, or β-actin overnight at 4 °C, followed by HRP-conjugated secondary antibody for 2 h at room temperature. Protein bands were visualized using an ECL detection kit. Normalization of total protein expression was carried out using β-actin as control.

### 4.10. Data Analysis

Data were expressed as mean ± SEM. One-way analysis of variance (ANOVA), followed by Dunnett’s *post hoc* test were used to determine statistical differences. A *p*-value of less than 0.05 was considered statistically significant.

## 5. Conclusions

In conclusion, the present study demonstrates that DNJ can increase hepatic insulin sensitivity via strengthening insulin-stimulated PKB/GSK-3β signal pathway and modulating glucose metabolic enzymes in *db*/*db* mice. Moreover, DNJ can improve lipid homeostasis and attenuate hepatic steatosis in *db*/*db* mice.
